# Assessment of methods for evaluating structural stability of cell envelope fragments in hypersaline brines as biosignatures of ancient microbial life

**DOI:** 10.1038/s41598-025-11211-7

**Published:** 2025-08-06

**Authors:** Lucas Bourmancé, Sébastien Brûlé, Bertrand Raynal, Adrienne Kish

**Affiliations:** 1 Unité Molécules de Communication et Adaptation des Microorganismes (MCAM), Muséum National d’Histoire Naturelle (MNHN), CNRS, Paris, France; 2 Institut Pasteur, Université Paris Cité, Plateforme de Biophysique Moléculaire, Paris, France

**Keywords:** Chaotropicity, Brines, Halophile, Hypersaline, *Halobacterium salinarum*, NanoDSF, AUC, DSC, Protein structural stability, Biochemical assays, Protein folding, Biological techniques, Archaea

## Abstract

The study of biomolecular stability of proteins and lipids in extreme saline environments is critical for understanding the preservation of potential microbial biosignatures of ancient life on Earth and other planetary bodies, including Mars. In this study, we evaluate the compatibility of several analytical techniques, Nano-Differential Scanning Fluorometry (NanoDSF), Analytical Ultracentrifugation (AUC), and Differential Scanning Calorimetry (DSC) with hypersaline brine analogues of Early Earth and Early Mars conditions. Using the halophilic archaeon *Halobacterium salinarum* as a model, we examine the structural stability of proteins within cell envelope fragments from dead cells, focusing on their preservation potential in complex brines. The results reveal significant technical challenges in studying macromolecules in high-salinity environments, including crystallisation during DSC and NanoDSF, viscosity-related artifacts in AUC, and reduced fluorescence signals in NanoDSF due to the low tryptophan content of membrane proteins from halophilic archaea. Nevertheless, NanoDSF proved useful for analysing multi-protein systems and DSC may be applicable using new generation technology, while AUC showed limited applicability under extreme saline conditions. These findings provide crucial insights into the methodologies for studying the stability of halophilic biomolecules in brine environments and the limitations of current techniques in extreme settings.

**Supplementary Information:**

The online version contains supplementary material available at 10.1038/s41598-025-11211-7.

## Introduction

On Earth, halophilic microorganisms have been found within NaCl (halite) crystals, inhabiting microdroplets of the fluid trapped within the crystalline matrix referred to as fluid inclusions^1^. These are often archaea closely related to *Halobacterium*^1^ and while the precise age of the microorganisms found in halite is estimated to be up to millions of years old^2,3^it remains uncertain. However, it is known that preserved biomolecules and cellular structures, particularly of dead cells, can be found within inclusion^4–6^. The haloarchaeal cell envelope fragments, composed of membrane lipids, membrane proteins, and cell wall surface layer (S-layer) proteins, constitute biosignatures of interest as the largest cellular macromolecular complex of these prokaryotic cells. Lipids are typically favoured for preservation over an extended time due to their stable hydrocarbon structure^7,8^. However, hypersaline environments may provide a more favourable environment for cell envelope protein preservation. In addition, the unique adaptations of halophilic proteins (increased number of acidic amino acid residues on protein surfaces, smaller hydrophobic patches, and salt bridges) together with the relatively protective environment of the lipid membrane can positively contribute protein preservation in the cellular remains of dead halophilic archaea. Brines inclusions are of particular interest for biosignatures detection due to their distinctive properties, such as low dissolved oxygen levels, higher viscosity, and specific ionic compositions that contribute to the preservation of biomolecules^9–12^. Additionally, they can be isolated from external environmental influences for up to millions of years^13,14^providing a more stable microenvironments for biosignatures from dead cells.

In this context, investigating the effects of various salts on the stability and preservation of both isolated proteins and cell envelope structures under relevant environmental conditions is therefore essential.

The study of biosignature stability in brine inclusions also hold significant exobiological interest, as hypersaline environments are present not only on Earth but also on various extraterrestrial bodies, including Mars and the icy moons Enceladus and Europa^15,16^. While terrestrial brines are typically characterized by a dominance of sodium and chloride ions, Martian brines are largely composed of magnesium, iron, and sulphate ions. These compositional differences arise from the unique evolutionary and geological histories of Earth and Mars, influencing the types of salts that precipitate and the ionic strength of their respective brines. Such differences in brine composition can greatly influence biosignature preservation through chaotropic and kosmotropic effects, referring to their disruptive or rigidifying effects on macromolecular structures, respectively^17–19^. Chao/kosmo-tropic effects have been previously studied^17–25^; however, it was done with simplified systems (single salt solution at sub-saturated concentrations, single protein sample) rather than natural, complex brines or intricate samples that may be encountered within fluid inclusions.

Studying microorganisms and their biomolecules in high-salinity environments requires developing and optimizing new methods, as conventional laboratory techniques are often unsuitable with brine concentrations. These incompatibilities include clogging effects (high concentration, viscosity)^26,27^side reactions (precipitation)^28,29^ and signal interferences (UV absorption, quenching effects)^30,31^. Only a limited number of techniques have been successfully validated for studying protein structural stability under hypersaline conditions. Investigations are, thus, necessary to identify techniques with potential compatibility, accompanied by extensive optimization to ensure their efficacy in hypersaline brines with different compositions, including both chaotropic and kosmotropic salts.

Circular dichroism (CD) has been employed in several studies to monitor specific structural changes (β-sheets and α-helices composition) in individual proteins from halophiles, demonstrating its effectiveness in characterizing secondary structural content, even in the presence of high salt concentrations^24,32,33^. However, CD has primarily been applied in simplified experimental contexts, typically involving single salt solutions and/or single protein samples. Additionally, CD analyses are difficult to apply to brines with a high chloride concentration as it absorbs in the far-UV range^31^.

In comparison, differential scanning calorimetry (DSC) has been utilized to determine the melting temperature (Tm) and thermodynamic properties of proteins at high single salt solution concentrations conditions^33^. Nano-differential scanning fluorimetry (NanoDSF), like DSC, can be used for analyses of mixed proteins samples. NanoDSF has shown promising results in assessing the thermal stability of halophilic proteomes across a range of saline conditions using soluble (cytosolic) proteins^23^. The use of NanoDSF and/or DSC is of significant interest as they provide broad data on different macromolecular levels, respectively proteins and proteins/lipids, providing more general information effect of salt of cell envelope structural overall stability.

In our previous study^12^NanoDSF analysis of complete cell envelope fragments incubated in different brines provided with first clues regarding chaotropic and kosmotropic effects but also shed light on the complexity of rending traditional methods compatible with hypersaline conditions. Here we use the same analogue brine solutions, to assess the compatibility of widely-used techniques for characterizing the structural stability of cell envelope proteins of dead halophilic microorganisms as analogue biosignatures. Cell envelopes and proteins extracted from cells of the well-characterized model haloarchaeon *Halobacterium salinarum* were employed, along with brines representative of early Earth and early Mars. The limitations and effectiveness NanoDSF, Analytical Ultracentrifugation (AUC), and DSC were compared using these systems to investigate the chao/kosmo-tropic effects of ionic composition on the structural stability of both proteins and lipids found within cell envelope fragments as biosignature of ancient life. Method compatibility with both a mixed composition macromolecular structure and a purified membrane protein (bacteriorhodopsin) was additionally explored using NanoDSF. Taken together, these results highlight methodological challenges for evaluating chaotropic and kosmotropic effects on microbial biosignatures in conditions simulating complex natural brine systems.

## Methods and results

### Experimental system

#### Biological material

*Halobacterium salinarum* was selected as a model system as it is closely related to isolates found in natural halite^1^. *Hbt. salinarum* NRC-1 was obtained from Dr. Caryn Evilia (Idaho State University). Five biological replicates of *Hbt. salinarum* NRC-1 were incubated from pre-cultures in Complex Medium+ (CM+: 4.28 M NaCl, 81 mM MgSO_4_.7H_2_O, 27 mM KCl, 10 mM trisodium-citrate.2H_2_O, 1% (w/v) peptone Oxoid^®^ LP0034, 0.5 (v/v) 100% glycerol, trace element solution: 6.3 µM FeSO_4_.7H_2_O, 1.5 µM ZnSO_4_.7H_2_O, 2.19 µM MnSO_4_, 4 nM CuSO_4_.5H_2_O, pH adjusted to 7.4) at 42 °C, 220 rpm in the dark (to mimic a fluid inclusion environment) until they reached stationary phase (OD600 = 1.3–1.4). To obtain cell envelope fragments for use as model biosignature structures, cells were first harvested by centrifugation at 10,000 x g for 10 min and the cell pellet resuspended in 10 mL of Basal Salt Solution (BSS: CM + without organics). Cell lysis was achieved by a single freeze/thaw cycle using liquid nitrogen. As *Hbt. salinarum* cells are highly polyploid, each sample was treated with 4 mg of DNAse 1 (DN25, Merck) for 30 min at 37 °C using a Tube Revolver (force 15) to remove DNA.

Raw cell envelope fractions were harvested by centrifugation 20,000 x g for 2 h at 4 °C and washed using three successive ultracentrifugation cycles (70.1Ti Rotor, Beckman) at 100,000 x g, 30 min, 4 °C with 10 mL of fresh BSS per cycle. The resulting cell envelope pellets were then resuspended in 1 mL Tris-HCl buffer, pH 7.4 (hereafter referred to as Tris buffer), and any remaining cytosolic contaminants removed using a 20–55% sucrose gradient with 5% increments in Tris buffer and centrifugation at 80,000 x g for 15 h at 10 °C. The red, carotenoid-bearing cell envelope fractions were collected and washed three times in Tris buffer by centrifugation at 229,600 x g, 4 °C for 1 h. The purified cell envelope fractions were resuspended in 2 mL of Tris buffer using an ice-cold ultrasonic bath (Advantage Lab, AL-04-04) for 5 min. Proteins were quantified by bicinchoninic acid (BCA) assay (Pierce™ BCA Protein Assay Kit, ThermoFisher Scientific), with 125 µg aliquots of cell envelope lyophilized and stored at -20 °C until use.

Complementary tests were performed using an isolated membrane protein as a simplified model system rather than complete cell envelope fragments. This approach allowed conclusions to be drawn about the compatibility of each method tested with hypersaline brines separately from the complexity of the mixed lipid and protein cell envelope structure, while using a relevant halophile protein model. For these, bacteriorhodopsin was selected. Bacteriorhodopsin (bR) is a pigmented membrane protein that functions as a light activated H^+^ pump as part of the so-called ‘purple membrane’^34^ allowing *Hbt. salinarum* to generate ATP through non-oxygenic phototrophy under low-oxygen, high-luminosity conditions^35^. Therefore, to induce bacteriorhodopsin production, *Hbt. salinarum* was cultivated in CM + media in non-baffled Erlenmeyer flasks under constant illumination 106.8 µmol.m^− 2^.s^− 1^. Flasks were sealed with 3 layers of parafilm. Head space was reduced by filling the flask to a 1:2.5 ratio to allow a semi-anoxic environment after initial growth to the expositional phase and oxygen depletion. After growth, cells were harvested by centrifugation (10,000 x g for 10 min) and resuspended in MQH_2_O causing osmotic shock and cells lysis due to the high internal salt concentration of *Hbt. salinarum* cells as part of the salt-in homeosmotic strategy. After DNAse treatment, bR was extracted following previously established protocols^36,37^. Briefly, a 20% (w/v) potassium phosphate solution and a 24% (w/v) polyethylene glycol (PEG) 8000 solution were prepared. Subsequently, 10 mL of each solution was added to 5 mL of the lysed cell extract. After 1 h of mixing using a Tube Revolver (Force 5), tubes were centrifuged 4,000 g at 4 °C for 30 min, and bacteriorhodopsin extract was collected at the interphase (Supplementary Fig. 1). The purple membrane extract was then washed three times in MQH_2_O.

Following preparation of both single protein (bR) and cell envelope fragments, brines were selected to mimic various Early Earth and Mars environments from different geographical regions and geological periods (Table 1). These complex brines were selected to assess the compatibility of different methods with complex natural brines^13,14,38^. The pH of each solution was not adjusted to preserve conditions more closely resembling the natural environment being modelled. As the brines were supersaturated, the solutions were prepared in borosilicate bottles by first adding the required salts to MQH_2_O at approximately 20% of the final desired solution volume. The mixture was continuously stirred with a magnetic stirrer for 1 h at room temperature, then the volume was completed with MQH_2_O before incubation for 5 days at 30 °C to equilibrate the liquid and solid phases. Finally, each bottle was sealed with three layers of parafilm to avoid evaporation and stored in the dark at room temperature.

#### Brine compositions

To investigate the effects of brines composition on complex macromolecular biosignatures, 125 µg of *Hbt. salinarum* cell envelope extracts were incubated in 500 µL of each brine shown in Table 1 using 1.5 mL microcentrifuge tubes. The brines used in this study were selected as analogues of terrestrial and Martian environments to investigate the effects of different composition on structural stability. An ice-cold ultrasonic bath was used to homogenize the solutions. The tubes were sealed with parafilm and incubated in the dark at 18 °C for 65 h prior to analyses by either AUC or DSC.

While all the brines shown in Table 1 were tested, the ionic composition of brine M3 was found to be incompatible with all methods used in this study, such that no conclusion could be drawn for this brine. This demonstrates the critical nature of brine composition, not only for biosignature preservation but also in the limitation of analytical methods to measure potential preservation. Therefore, the following sections will focus only on the three remaining Martian-type brines (M1, M2, M4), and four terrestrial-type brines (E1, E2, with BSS and BSS-LS as controls).

Stability of cell envelope fragments and component proteins derived from *Hbt. salinarum* were evaluated using two methods for global stability macromolecular structures (AUC and DSC) targeting different biochemical characteristics as described below, as well as one method (NanoDSF) specific to the higher order structure of proteins. For all chao/kosmo-tropicity analysis performed in this study, cell envelope fragments extracted from *Hbt. salinarum* cells cultured under optimal growth conditions in CM + growth medium were compared to the ‘control’ BSS brine (CM + without carbon sources). Brines that would destabilise or stabilize those conditions would be considered respectively as chaotropic and kosmotropic.

**Table 1:** Brine compositions. BSS Brine serves as the control Brine, as its composition is identical to the growth medium used for *Hbt. Salinarum* without carbon sources. BSS-Low salt (BSS-LS) is formulated identically to the BSS Brine, except for a reduced NaCl concentration of 2.9 M, which represents the minimum salt concentration required to support the growth and survival of *Hbt. Salinarum*. Chao/kosmo-tropicity measurements were taken from^12^. Negative values indicate chaotropic activity, positive values indicate kosmotropic activity.

Brine composition (mol.L^− 1^)	Early mars				Early earth		Controls	
	Type I (Nakhla Martian meteorite)	Type II (Merridiani plunum)	Type III (same as II but more acidic)	Type IV (Phoenix Lander site North Pole)	San Andres Formation (Guadaluoian, 274–272 Ma)	Bresse Bassin (Eocene, 36-34 Ma)		
	M1	M2	M3	M4	E1	E2	BSS	BSS-LS (low salt)
NaCl	1.27	2.27	1.36		5.56	2.66	4.28	2.89
KCl	3.78	1.33	1.14		0.27	0.53	0.27	0.27
MgCl_2_.6H_2_0		1.15	3.70		0.18	1.33		
MgSO_4_.7H_2_0		2.55					0.81	0.81
HCl		0.39	0.11					
Tri-sodium citrate.2H_2_0							0.13	0.13
CaCl_2_						0.60		
KHCO_3_	2.24							
FeSO_4_.7H_2_0			2.31					
FeCl_2_.4H_2_0			0.99					
Na_2_SO_4_					0.80			
Mg(Cl0_4_)_2_				4.16				
pH	8.3	1	0.5	4.6	4.68	5.67	7.4	7.4
Chao-kosmotropicity compared to H_2_O	− 87.15	6.23	410.85	< 410.85	− 43.58	0	− 43.58	− 37.35
Chao-kosmotropicity compared to BSS	− 43.58	37.35	454.43	< 454.43	0	43.58	–	6.23

### Analytical ultracentrifugation (AUC)

AUC is a technique used to study the size, shape, molecular weight, and interactions of macromolecules in solution. Samples are subjected to high centrifugal forces, causing particles to sediment according to their mass, shape, and buoyancy. The sedimentation process is monitored in real-time using optical systems, typically through absorbance (light absorption of molecules) or interference (fringe shifts: refractive index differences between molecules and solvent) detection. Macromolecules are characterized by measuring their sedimentation coefficient. This coefficient is a measure of the rate at which particles sediment under the influence of centrifugal force. It is defined as the ratio of the sedimentation velocity of the particle to the applied centrifugal field (ω^2^r), where ω is the angular velocity of the centrifuge rotor and *r* is the radial distance from the axis of rotation to the particle. The sedimentation coefficient is typically expressed in Svedberg units (S), where 1 Svedberg (1 S) = 10^− 13^ seconds. The value of S reflects the size, shape, and buoyancy of the particle in the solvent, providing critical insights into the particle’s molecular weight and conformational state. For samples with same density, a larger sedimentation coefficient indicates faster sedimentation, which is associated with bigger mass or more compact molecules, while smaller values correspond to slower sedimentation of smaller mass or more elongated particles. AUC enables the generation of specific sedimentation distribution profiles for each sample, thereby allowing a detailed assessment of the effects of various brines on cell envelope biomolecules, including proteins and lipids. These profiles provide valuable insights (heterogeneity, aggregation, shape, conformation, molecular mass, of macromolecules) into the structural and stability changes induced by the brines on the molecular components of the cell envelope.

Sedimentation velocity experiments were carried out at 20 °C in an Optima AUC (Beckman-Coulter) equipped with double UV and Rayleigh interference detection. Samples were prepared and spun using an An50Ti rotor and 12-mm double-sector epoxy centrepieces. The partial specific volume of the sample was set at 0.730 mL.g^− 1^. Viscosity was measured using a viscosizer TD (Malvern) and density was measured using a DNA 5000 M (Anton Paar). Interference profiles were recorded every 3 min. Sedimentation coefficient distributions [c(s)] were determined using the software Sedfit 15.5^39^.

Initial tests were conducted using cell envelopes incubated in BSS as a control and the M4 brine. The M4 brine was selected due to a notable color change from red, induced by the lipid carotenoid pigment bacterioruberin, to orange observed during the incubation process, indicating potential structural alterations in the cell envelope biomolecules.

**Fig. 1:**
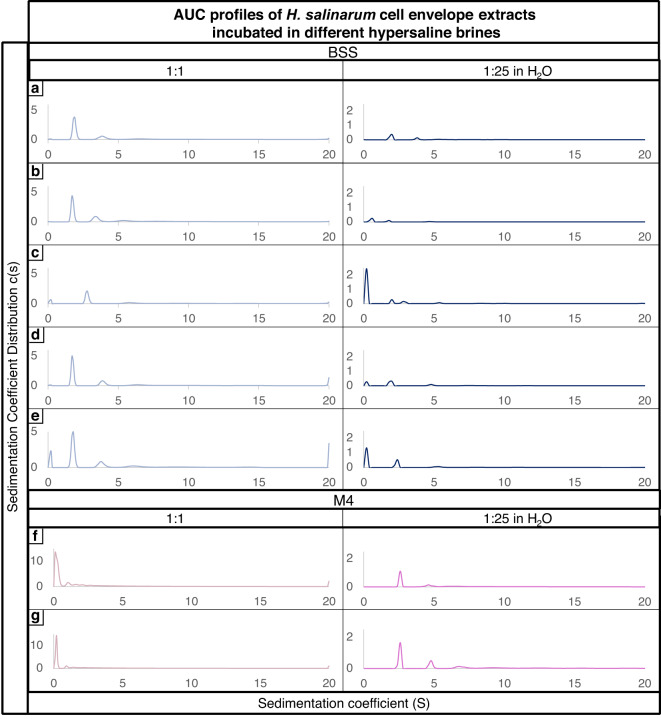
AUC profiles of *Hbt. salinarum* cell envelope extracts incubated in BSS and M4 brines (S = sedimentation coefficient; c(s) = sedimentation coefficient distribution). (**a**–**e**) are the 5 biological replicates of cell envelope extract incubated in BSS, and (**f**,**g**) are the 2 biological replicates of cell envelope extract incubated in M4. Each peak is attributed to a different macromolecular species found in the sample. Using pure solutions led to viscosity and density issues during the centrifugation process, thus a 1:25 in MQH_2_0 was utilized. However, it is hypothesized that this dilution may have induced restructuring effects that masked the true behaviour and influence of the brine, potentially compromising the accuracy of the experimental outcomes.

AUC sedimentation profiles were reproducible across biological replicates for both BSS and M4 brines, with clear distinctions between the two conditions (Fig. 1). For cell envelope extracts incubated in BSS brine, two primary species were identified, with sedimentation coefficients of approximately 1.6–1.7 S and a less abundant species around 3.5 S. In M4 brine profiles, one major peak was observed around 1 S. This suggested that the M4 brine had an impact on the structure of the cell envelope fragment, potentially inducing aggregation and overall degradation.

However, the high viscosity of the brines raised concerns about whether the observed differences were due to genuine structural modifications of the cell envelope or potential non-ideal behaviour resulting from the composition of the brines. To address this issue, the samples were diluted 1:25 in MQH_2_O water to reduce viscosity, which resulted in improved sedimentation and the detection of additional species. However, the overall sedimentation profile between the two brines did not show any major differences, as would be expected based on their divergent properties. Dilution with pure water therefore may mask the true effects of brines on cell envelope extract structural stability. Moreover, a previous study on salt effects (NaCl, MgCl_2_ and CaCl_2_) on proteins revealed secondary structure modification with changes in alpha helices and beta sheets^24^. It is likely that the primary differences between the BSS and M4 brines may mostly involve secondary structure alterations without change in mass and overall shape, which cannot be detected by AUC.

As a result, it was concluded that AUC is not the most suitable technique for experiments involving high-salt buffers due to the potential for viscosity-related artifacts and the limitations of the technique in detecting subtle conformational changes. Therefore, other techniques less sensitive to viscosity and density effects were tested, including DSC.

### Differential scanning calorimetry (VP-CAP-DSC, Malvern)

DSC is a thermodynamic technique used to measure the heat flow associated with phase transitions or thermal denaturation of biomolecules as a function of temperature. The instrument measures the differential heat flow between the sample (here cell envelope extracts) and a reference, typically the same buffer used for the sample or in this case brine. During the run, the heat required to raise the temperature of the sample changes smoothly. However, when the sample undergoes a phase transition, the amount of heat absorbed or released changes suddenly producing an endothermic peak (most common for biomolecules) or an exothermic peak, respectively.

Here, DSC was carried out on a Microcal VP-capillary DSC (Malvern-Panalytical). The capillary was filled with 190µL of cell envelope extracts in each of the brine solutions. The samples were scanned from 20 °C to 110 °C with a scan rate of 1 °C/min, first with repeated scans of each brine solution to ensure a stable brine baseline, and then with the cell envelope containing solutions.

**Fig. 2:**
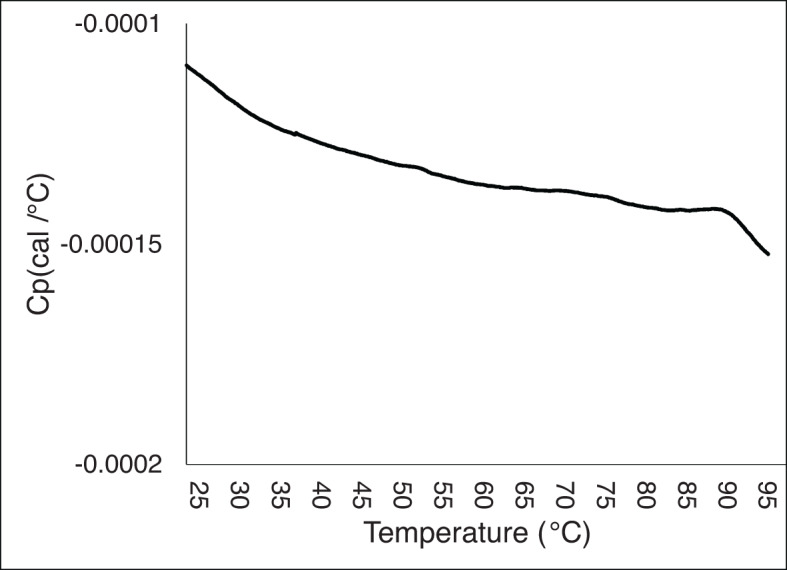
DSC profile of *Hbt. salinarum* cell envelope fragments incubated in BSS. The downward tendency observed reveals an exothermic event that can be attributed to the crystallisation process, obscuring any features related to changes in the structural stability of biomolecules.

However, these tests were inconclusive due to the crystallisation of the hypersaline solution in the instrument capillary with increasing temperatures. This prevented accurate data acquisition (Fig. 2) and was confirmed by visual observations of crystallisation after the analytical run.

To minimize crystallisation process at high temperatures, NanoDSF was instead employed. This method utilizes individual capillaries for sample deposition, which reduces the impact of crystallisation on the performance of the instrument.

### Nano-differential scanning fluorometry

NanoDSF was used to monitor protein folding/unfolding processes across a temperature gradient by tracking the intrinsic fluorescence of tryptophan residues (Trp) with excitation at 280 nm and emission measured at 330 nm and 350 nm. During thermal denaturation, the fluorescence emission peak may shift from 330 nm (folded state) to 350 nm (unfolded state). The ratio of fluorescence intensity at 350 nm to 330 nm (350/330) was recorded as a function of increasing temperature. The melting temperature (Tm), defined as the temperature at which 50% of the protein population is unfolded, was determined by calculating the first derivative of the 350/330 ratio. The Tm value provides a comparative measure of the thermal stability of the protein sample.

This method had previously been successfully applied to evaluate the effects of salts on cytosolic (soluble) proteins from *Hbt. salinarum*^23^. We recently validated this technique for use with *Hbt. salinarum* cell envelope fragments (insoluble proteins)^12^. However, the results obtained for cell envelope stability in certain mixed-salt solutions such as the M1 brine were difficult to interpret due to the absence of clear transition of fluorescence emission. Therefore, a sample complexity reduction approach was evaluated here to determine if it could improve data clarity and interpretation.

Cell envelope extracts were replaced by aliquots of isolated bR proteins (125 µg), following the same brine incubation protocol. Resuspended bR extracts were transferred into quartz capillaries, which were subsequently loaded into the NanoDSF instrument for thermal stability analyses.

**Fig. 3:**
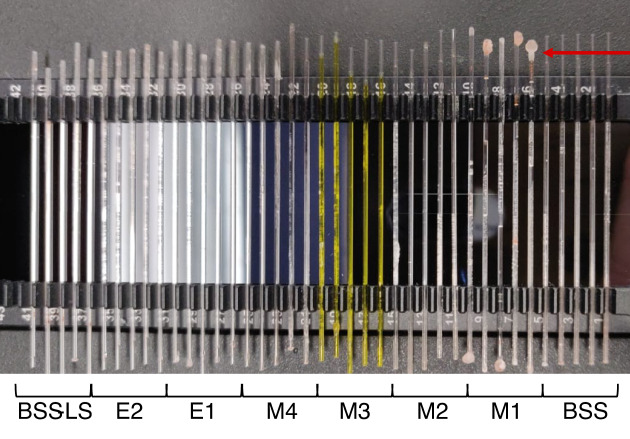
Nano-DSF glass capillaries filled with different brines. Crystallisation processes can be observed at the extremities of the capillaries. The red arrow indicates extensive crystallisation at the extremity of the capillaries containing the M1 brine. However, crystallisation was not limited to the M1 brine but to all of them. It could be observed both at the extremities and within the capillaries.

**Fig. 4:**
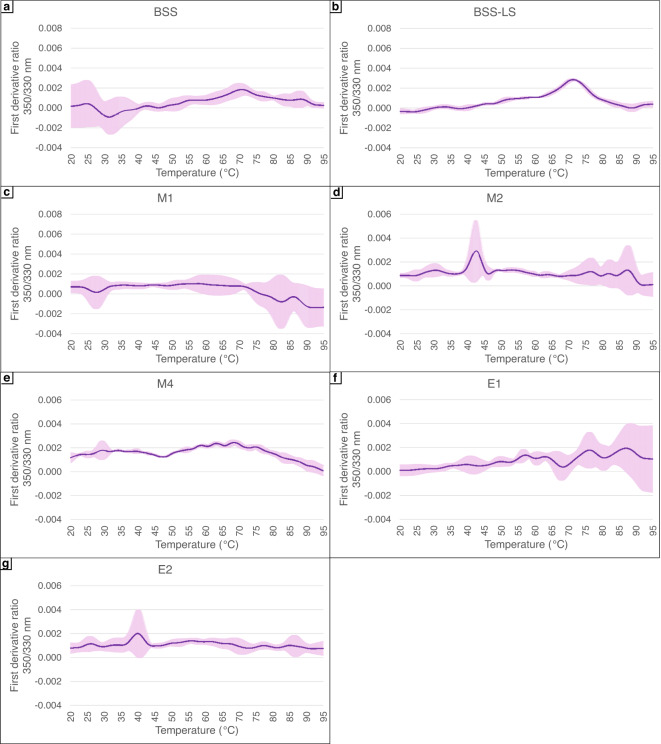
NanoDSF first derivative of 350/330 nm ratio of bR samples. Temperature gradient was performed from 20 °C to 95 °C at a rate of 1 °C per minute. Pink areas represent the standard deviation calculated from five biological replicates. (**a**) to (**g**) show the results for the different brines, as indicated.

During initial testing, it was observed that hypersaline solutions within the capillaries crystallised at elevated temperatures (Fig. 3). To limit this issue, the quartz capillaries were sealed using capillary sealing paste (PR-P001, Nanotemper). This modification enabled successful measuring of NanoDSF curves (Fig. 4). A decrease in Tm relative to the BSS control was interpreted as a chaotropic effect, indicating that less thermal energy was required for protein unfolding due to destabilization of the protein structure. Conversely, an increase in Tm was taken as evidence of a kosmotropic effect, reflecting increased protein stability and more rigid folding, which require higher temperatures to induce unfolding.

Among the brines tested, only the bR samples incubated in the M2, E2, and BSS-LS brines exhibited a minor peak corresponding to the thermal transition from the folded to unfolded state (Fig. 4.b, d and g). The absence of detectable thermal transitions in the remaining brines can be interpreted as either the absence of a typical unfolding event or the test conditions prevented the detection of transitions. However, these results can be compared to the agar proxy method previously tested with the brines (Table 1) and properly interpreted. Bacteriorhodopsin samples in E2 and M2 brines showed thermal transitions at 40–45 °C indicating a more chaotropic effect than samples incubated in BSS-LS, which displayed a kosmotropic effect with a recorded transition at 70–75 °C.

Compared to cell envelope fragments^12^the transitions recorded here for purified bR were generally reduced. Only the E2 and M2 showed better transitions when biological sample complexity was reduced. The results of the remaining brines were either comparable to those observed with cell envelope extracts or demonstrated less pronounced transition peaks. This was particularly evident in the control solutions BSS and BSS-LS for which clear transitions were observed with the cell envelope extracts around 80 °C, whereas here the transition peaks were drastically diminished. Specifically, the transition amplitude was reduced in the BSS-LS solution and almost completely flattened in the BSS solution (Fig. 4a and b). Additionally, due to compositional and structural change compared to other proteins, a decrease in Tm was notable with bR samples. Finally, consistent with results obtained for cell envelope fragments in the same brines, no transitions were observed for bR in brines M1 and M4. The respective extreme kosmotropic and chaotropic nature of M1 and M4 brines relative to BSS likely increased (M1) and decreased (M4) the transition temperature of both complex and simple biological samples. This implies that bR samples were already denatured in the M4 brine and more rigidified in the M1 brine. The latter could be evaluated using nanoDSF machine reaching higher temperature, although extensive crystallisation might interfere with the analysis.

The reduced NanoDSF transition peak intensities can likely be attributed to the fact that haloarchaeal proteins, in particular cell envelope proteins, typically contain fewer tryptophan residues for a given concentration. This adaptation serves to lower the overall hydrophobic content and maintain structural stability in hypersaline environments. As a consequence, the proteins intrinsic fluorescence signal is reduced resulting in less pronounced thermal transition profiles during analysis. Therefore, simplifying the system to a single protein, although allowing for the detection of a more specific signal, is not a viable technical approach when employing NanoDSF with halophilic cell envelope proteins. However, the simplification could be an effective analytical strategy for cell envelopes containing membrane proteins with a higher proportion of tryptophan residues.

## Discussion

Chaotropicity has received great interest over the last years due to its impact on habitability and has been suggested to be a limit of life^40–42^. However, most studies working either on chaotropic effects on the growth of microorganisms or on macromolecular structural stability have been limited to either single salt solution, low concentrations and/or soluble proteins. The complexity of natural brines demands the screening of methods to establish a reliable framework to investigate the chaotropic effect in the lab but also for on-site missions. In this context, this study tested the compatibility of three methods (NanoDSF, AUC, and DSC) with hypersaline brines analogous to natural environments on Early Earth and Mars, assessing their chao/kosmo-tropic effects on complex cell envelopes or simple bacteriorhodopsin extracts from *Halobacterium salinarum* as biosignatures of dead cells.

The incompatibility of any method with one of the brines tested, the M3 brine, demonstrated the importance of the compositions of both the brine and the biological material in question. The high iron concentration present in the M3 brine interacted with cell envelope proteins resulting in mineralization. Previous studies have highlighted the interaction between metal ions such as iron and copper with the prokaryotic cell wall surface layer (S-layer) proteins due to the presence of negatively charged residues^43–45^. Salt-adapted proteins such as *Hbt. salinarum* cell envelope proteins are enriched with such residues, potentially leading to conformational changes, mineralization, and subsequent incompatibility with the analytical methods in the presence of high iron concentrations. Therefore, the ionic composition including both salts and metals as well as the amino acid composition of the proteins need to be takin into consideration for analyses of microbial biosignatures in brines.

NanoDSF has previously been employed to investigate the stability of the soluble proteome of *Hbt. salinarum* under varying salinity and salt conditions^23^ as well as the insoluble proteins of the cell envelope^12^. While there are relatively fewer proteins to analyse in a cell envelope compared to a cytosolic proteome, the analytical complexity for NanoDSF was simultaneously increased due to the decreased number of tryptophan residues in cell envelope proteins compared to cytosolic proteins, thereby reducing the detectable signal. Here, even though chao/kosmo-tropic effect were still detectable, we confirm the hypothesis that the relative quantity of Trp residues is a more critical factor than sample macromolecular complexity for the success of NanoDSF analyses. While increasing the concentration of the sample could potentially provide with clearer signal, solubility and aggregation effects would bias the results^46^. To avoid the limitations of NanoDSF for low-Trp protein samples and to include cell envelope lipids in the cell envelope structural stability analysis, the techniques of AUC and DSC, which do not rely on fluorescence, were assessed.

Although AUC is a well-established technique for the detailed characterization of complex biological samples^47^it was ultimately found here to be incompatible with hypersaline solutions. The high viscosity and density of complex brines rendered the interpretation of AUC data inconclusive, as observed differences could not be reliably attributed to chaotropic effects. Attempts to improve the centrifugation process by diluting the solution with MQH_2_O after incubating cell envelope extracts in the brines to improve the centrifugation process did not result in a sedimentation distribution profile with a clear brine signature. In addition, the timeframes typical for AUC experiments, which can extend over several hours, can be too long to follow the rapid effects of certain brines on protein-bearing structures^12^. The challenges encountered during AUC highlight the importance of the physicochemical characterisation of the brines by measuring viscosity and density to avoid misinterpretations of the data.

In the case of DSC, solution crystallisation during the run truncated the analyses. To prevent temperature-induced crystallisation, an alternative approach could involve avoiding capillary-based DSC or any microfluidic DSC-system, thereby reducing the surface-to-volume ratio of the samples. As next-generation DSF instruments become more readily available, some may be found that are compatible with saturated brines. Of particular interests for improving data quality would be models that employ crucibles for sample deposition, and those that record the full emission spectrum at each temperature ramp allowing wavelength selection to better measure transition temperatures.

Overall, these results underscore the complexity of studying the thermal stability and structural behaviour of halophilic membrane proteins in hypersaline environments. Each technique has a limiting factor that must be considered for each brine and each type of biological material in question. NanoDSF is well-adapted to studying chao/kosmo-tropic effects of different brines on macromolecular structures, depending on the Trp content of the component proteins and preparation of the sample capillaries. DSC harbours great potential for detailed structural analysis of complex samples that should be pursued with the upcoming generation of instruments to avoid sample crystallisation during analytical runs. Such adaptations in experimental design and the integration of complementary techniques will be essential for advancing our understanding of protein and membrane structure behaviour in extreme salt environments, particularly for the study of biosignatures of past microbial life.

## Electronic supplementary material

Below is the link to the electronic supplementary material.

Supplementary Material 1

## Data Availability

The datasets used and/or analysed during the current study available from the corresponding author on reasonable request.
