# Supplementary material for: Assessment of methods for evaluating structural stability of cell envelope fragments in hypersaline brines as biosignatures of ancient microbial life
Source: Sci Rep. 2025 Aug 6;15:28677. doi: 10.1038/s41598-025-11211-7 (PMC12328702; doi:10.1038/s41598-025-11211-7)
Supplement: Supplementary file 1 — Supplementary Material 1 [file 41598_2025_11211_MOESM1_ESM.pdf]

Supplementary Information

Supplementary Figure 1 : bR interphase after separation in PEG800 and potassium phosphate solutions.

Purple layer indicates the presence of extracted bacteriorhodopsin proteins.

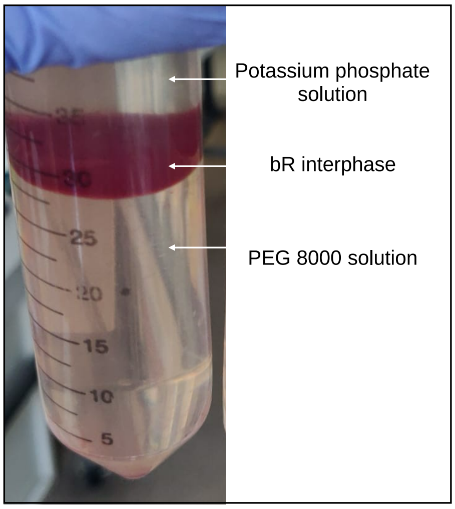

Supplementary Table 1: AUC major peaks list of cell envelope extracts incubated in Basal Salt Solution (BSS).

A dilution to 1:25 with MQH_2O was used to reduce viscosity of the brine for proper centrifugation.

Brine	Replicate	Major Peaks	Sedimentation Coefficient (S)	Sedimentation coefficient distribution c(s)	% of total species
BSS (1:1)	1	1	1.876	1.1571	20.41
		2	3.927	0.3934	6.94
		3	6.823	0.2428	4.28
	2	1	1.741	1.08	19.72
		2	3.439	0.5042	9.15
		3	5.54	0.3102	5.63
	3	1	2.781	0.6338	5.4
		2	6.037	0.1843	1.572
		3	9.834	0.1327	1.13
	4	1	1.737	1.1737	16.13
		2	3.927	0.4036	5.54
		3	6.592	0.2442	3.35
	5	1	1.771	1.4514	29.02
		2	3.829	0.4638	9.27
		3	-	-	-
M4 (1:1)	1	1	1.114	0.6914	9.15
		2	1.688	0.4277	5.66
		3	2.74	0.346	4.58
	2	1	0.19	2.188	53.38

		2	0.984	0.2958	7.22
		3	1.632	0.3265	7.97
		4	2.333	0.2181	5.32
Brine	Replicate	Major Peaks	Sedimentation Coefficient (S)	Sedimentation coefficient distribution c(s)	% of total species
BSS (1:25)	1	1	1.938	0.1083	41.25
		2	3.793	0.0385	14.66
		3	5.338	0.0207	7.89
		4	7.477	0.0116	4.44
		5	8.902	0.0041	1.57
		6	10.353	0.0116	4.413
		7	12.871	0.0084	3.219
		8	15.341	0.0114	4.343
		9	18.641	0.0086	3.289
		10	25.463	0.005	1.89
	2	1	0.545	0.0701	44.67
		2	1.759	0.0252	16.07
		3	4.764	0.0129	8.21
		4	9.22	0.0093	5.93
		5	13.496	0.0042	2.7
		6	18.237	0.01	6.39
		7	22	0.0007	0.46
		8	24.295	0.0036	2.33
	3	1	2.04	0.0728	10.76

		2	2.845	0.0576	8.51
		3	5.356	0.025	3.70
		4	8.45	0.0177	2.61
		5	9.911	0.0127	1.88
		6	19.82	0.0079	1.17
	4	1	1.91	0.1147	42.89
		2	4.804	0.0363	13.58
		3	7.762	0.0231	8.65
		4	11.504	0.0031	1.171
		5	16.878	0.0088	3.28
		6	21.072	0.0058	2.19
		7	25.121	0.0031	1.15
		8	29.064	0.0015	0.55
	5	1	2.356	0.1405	27.29
		2	5.312	0.0426	8.28
		3	8.148	0.019	3.7
		4	11.049	0.0193	3.75
		5	14.939	0.0167	3.25
		6	19.63	0.0051	0.99
		7	22.898	0.0009	0.17
M4 (1:25)	1	1	2.602	0.2532	37.38
		2	4.692	0.0927	13.69
		3	6.066	0.0458	6.73
		4	7.004	0.0477	7.04
		5	8.793	0.019	2.8

		6	13.609	0.0554	8.18
		7	22.372	0.0265	3.92
		8	33.919	0.0088	1.29
		9	39.097	0.0115	1.7
		10	45.181	0.0063	0.93
	2	1	2.573	0.3827	31.17
		2	4.781	0.1701	13.85
		3	7.003	0.1265	10.3
		4	9.519	0.1102	8.97
		5	12.58	0.0867	7.06
		6	15.67	0.0575	4.69
		7	19.078	0.0566	4.61
		8	22.735	0.0421	3.43
		9	32.101	0.0249	2.03
		10	37.209	0.0361	2.94
